# Effects of sensory substituted functional training on balance, gait, and functional performance in neurological patient populations: A systematic review and meta-analysis

**DOI:** 10.1016/j.heliyon.2021.e08007

**Published:** 2021-09-17

**Authors:** Peter Lynch, Kenneth Monaghan

**Affiliations:** a Clinical Health and Nutrition Centre (CHANCE), School of Science, Institute of Technology (IT) Sligo, Ireland; b Neuroplasticity Research Group, Clinical Health and Nutrition Centre (CHANCE), School of Science, Institute of Technology (IT) Sligo, Ireland

**Keywords:** Sensory substitution, Neurorehabilitation, Neuroplasticity, Neuropsychology, Balance, Gait, Systematic review, Meta-analysis

## Abstract

**Introduction:**

Sensory Substitution (SS) is the use of one sensory modality to supply environmental information normally gathered by another sense while still preserving key functions of the original sense.

**Objective:**

This systematic literature review and meta-analysis summarises and synthesise current evidence and data to estimate the effectiveness of SS supplemented training for improving balance, gait and functional performance in neurological patient populations.

**Methods:**

A systematic literature search was performed in Cochrane Library, PubMed, Web of Science, and ScienceDirect. Randomized controlled trials (RCTs) using a SS training intervention were included.

**Results:**

Nine RCTs were included. Outcome measures/training paradigms were structured according to the balance framework of Shumway-Cook and Woollacott: Static steady-state, Dynamic steady-state and Proactive balance. Meta-analyses revealed significant overall effects of SS training for all three outcomes, as well as self-assessment and functional capacity outcomes, with Dynamic Steady-State balance and ability of stroke survivors to support bodyweight independently on paretic side lower limb found to have had the largest statistical and clinical effects. Meta-analyses also revealed non-significant retention effects.

**Conclusion:**

This review provides evidence in favour of a global positive effect of SS training in improving Static steady-state, Dynamic steady-state and Proactive balance measures, as well as measures of self-assessment and functional capacity in neurological patient populations. Retention of effects were not significant at follow-up assessments, although no intervention met training dosage recommendations. It is important for future research to consider variables such as specific patient population, sensor type, and training modalities in order identify the most effective type of training paradigms.

## 1 Introduction

In 2016, neurological disorders were the leading cause of Disability-Adjusted Life-Years (DALYs) to be lost (276 million years) and second leading cause of mortality (9 million) globally [1]. In Europe, in 2017, neurological disorders accounted for over forty-one million DALYs lost and approximately two million deaths [2, 3]. Research by the World Health Organization has projected that in 2030, there will be a twelve percent increase in the number of global DALYs lost due to neurological disorders since 2005 [4]. This projection estimates that approximately seven percent of overall global DALYs lost, and over twelve million deaths per year, will be attributable to neurological disorders [4]. Stroke was reported as the leading cause of neurological disorder mortality and DALYs lost globally and in Europe in 2016 and 2017 respectively [2, 3], and is predicted to account for over half of all DALYs and mortality due to neurological disorders by 2030 [4]. In the United Kingdom alone, a 2020 projection estimates the number of stroke survivors will more than double over the next two decades [5]. The most common reported deficit induced by stroke, and most neurological disorders, is motor impairment, which can be described as loss or limitation of muscle control function or movement, or limitation in balance and mobility [6]. Loss of balance when mobilising is common for most neurological disorders, with approximately seventy percent of stroke survivors living at home reported to fall within a year of their stroke [7]. According to a Systematic Literature Review (SLR), approximately two-thirds of stroke survivors have initial balance and mobility deficits, and over thirty percent still cannot mobilise independently after six months [8]. The authors highlight that one of the key goals of neurorehabilitation is to improve mobility [8].

Neuroplasticity is defined as the ability of the Central Nervous System (CNS) to undergo structural and functional change in response to new experiences and stimuli [9]. Sensory Substitution (SS) is an intervention modality based on the principle of neuroplasticity. SS is a biofeedback modality in which one sensory system (e.g., hearing) is used to supply environmental information normally gathered by another sense (e.g. vestibular) [10]. SS is an intervention devised from the work of Neuroscientist Paul Bach-Y-Rita [11]. Bach-Y-Rita and his team first focused on SS neurorehabilitation by substituting compromised visual apparatus with tactile feedback in congenitally blind individuals to help them “see” through projected visual imagery [11]. Recent evidence provides physiological rationale for this work with functional Magnetic Resonance Imaging (fMRI) demonstrating occipital/visual cortex activity in blind people during nonvisual tasks such as Braille reading, or sensory discriminations of auditory or tactile stimuli [12]. Interestingly, an additional brain imagining investigation discovered that following a short period (5 days) of complete visual deprivation, the occipital cortices of sighted people began to process non-visual tactile stimulus [13]. This tactile processing was no longer present 24 h after blindfold removal [13]. The speed and dynamic nature of the observed changes suggests that normally inhibited or masked neuronal connections in the sighted are revealed by visual loss, and, represent rapid, early plastic changes, which presumably can lead, if sustained and reinforced, to slower developing, but more permanent structural changes [13]. This property of the CNS to adapt to sensory deprivation is the foundation of neurorehabilitation through SS. There is a seemingly small evidence base for investigation of SS in neurological disorders causing motor impairment, although the ability of the CNS to reorganise cortical functions after severe neurological disruption such as stroke has been explored in research [14].

The human brain interprets and integrates information from various sensory modalities into a complete representation of surrounding events, a function known as multisensory integration. Evidence suggests that multisensory processes appear to be largely preserved in many neurological disorders [15, 16]. According to a review by Bolognini and colleagues, the benefit of multisensory integration on the recovery of motor functions after neurological disruption has not been well established [17]. A SLR and Meta-Analysis (MA) by Gordt and colleagues [18] previously investigated the effects of SS devices on balance, gait, and function in neurological patients, but included healthy adults and other patient populations in the cohort analysed. The aim of this SLR and MA is to examine and estimate the effect of balance, gait and functional training supplemented by SS solely in neurological patient populations.

## 2 Methods

This review was conducted according to the Preferred Reporting Items for Systematic Reviews and Meta-Analyses (for protocols) (PRISMA-P) checklist [19]. PRISMA-P is intended to guide the development of protocols of SLRs and MAs evaluating therapeutic efficacy [19]. A computer-aided search of databases the Cochrane Library, ScienceDirect, PubMed and Web of science was conducted to search for relevant articles published in the English language. The literature search employed a combination of keywords and Boolean operators (AND or OR). No SS specific Medical Subject Headings (MESH) were found, and therefore a narrow search string using only *Intervention* keywords was used, in order to produce the most relevant research and preserve a concise scope as per the PRISMA-P checklist [19], and as used in previous literature [20]. Table 1 displays the search strategy employed.

**Table 1:** Search strategy.

Intervention
Sensory substitut∗
**OR**
Sensory augmentat∗

∗ = Truncation; i.e. search sensitive to articles that include any ending of root word.

SS and Sensory Augmentation were truncated and selected as *Intervention* specific search parameters as recent literature highlights this terminology to distinguish this specific modality of rehabilitative devices from non-substitution stimulation devices [21, 22]. No limit on date of publication was set. Where applicable, a ‘clinical trial’ filter was applied to further accumulate the most relevant research for this review.

Inclusion criteria for this SLR and MA was: (1) *participants*: Any patient population diagnosed with a neurological disorder affecting motor function, as confirmed by a neurologist/medical consultant; (2) *intervention*: SS and functional training; (3) *comparators*: conventional therapy/training, or placebo SS, or explicate control group; (4) *outcomes*: recognised measures of disability (to include motor/functional performance), impairment and handicap; and (5) *study design*: Randomised Controlled Trial (RCT). SS is provision of information to the brain that is usually in one sensory domain (e.g., visual information via the eyes and visual system) by means of the receptors, pathways, and brain projection, integrative and interpretative areas of another sensory system (e.g., information through the skin and somatosensory system) [23]. Neurological disorders are diseases of the CNS and Peripheral Nervous System (PNS) (i.e. the brain, spinal cord, cranial nerves, peripheral nerves, nerve roots neuromuscular junction, and muscles) [24].

The BMJ Framework for assessing Randomised Controlled Trials [25] was applied to assess study quality. Of the framework criteria, studies meeting 80% (16/20) or above were classified as High quality; 60% (12/20) as Moderate quality and those meeting below 12/20 as Low quality [25]. The Cochrane Risk of Bias Assessment Tool was also applied to included studies [26].

To structure data extraction and summary, the balance framework of Shumway-Cook and Woollacott [27] was used to classify training paradigms and outcome measures. According to the framework, balance is a complex composite of multiple body systems cohesively producing the ability to align different body segments to effectively control body position and movement [27]. As per the framework, balance can be classified as Static Steady-State (i.e., maintaining a steady position in sitting or standing), Dynamic Steady-State (i.e., gait), Proactive (i.e., anticipating a predicted disturbance such as crossing or walking around an obstacle), and Reactive (i.e., compensating a disturbance) [27].

In order to calculate the effect of SS training compared with controls, random-effects MA on the most frequently reported outcome measures for Static Steady-State, Dynamic Steady-State and Proactive balance measures were applied. No study reported on Reactive balance. Random-effects MA's were used as outcome measures estimating treatment effect varied across studies. Additional appropriate MA of outcome measures which were relatively consistent across the research were also applied. Effect size was calculated using Standardised Mean Difference (SMD) as varying outcome measures were used. SMD is calculated by $\frac{Difference\;in\;mean\;outcome\;between\;groups\;}{Standard\;deviation\;of\;outcome\;among\;participants}$ [28]. A positive SMD indicates improvements in favour of the intervention group. For statistical effect, a P-value of less than 0.05 indicates significance [28]. For clinical effect, SMD value can be classified as Small (SMD = 0.2), Medium (SMD = 0.5) and Large (SMD = 0.8) effect [29]. Heterogeneity was assessed using Cochrane's I^2^ test. As per Cochrane recommendations, thresholds for heterogeneity are:

- 0%–40%: might not be important;
- 30%–60%: may represent moderate heterogeneity;
- 50%–100%: may represent substantial heterogeneity [28].

According to the Cochrane recommendations, it is not recommended to test for funnel plot asymmetry when there are less than ten studies in a MA [28]. The MA's were performed using The Cochrane Collaboration Review Manager (RevMan) (Version 5.4) [26].

## 3 Results

### 3.1 Systematic review

#### 3.1.1 Study selection

**Figure 1:**
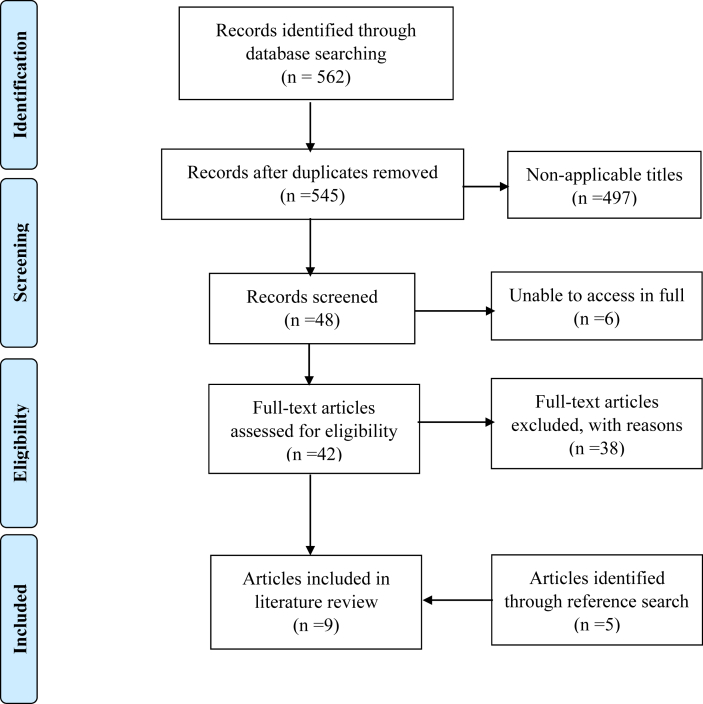
PRISMA flow diagram of article extraction process.

The search strategy yielded a total of 562 articles (see Figure 1). The literature search was conducted on 19/06/20. Study selection was performed by both authors (P.L., K.M.). In the case of disagreements, the articles were discussed by the authors in accordance to meeting inclusion criteria. Articles yielded from the search were screened by identifying any potentially relevant articles from the titles. Duplicates were first removed. Abstracts of all potentially relevant articles were screened and read. Case reports, letters, conference abstracts, posters and systematic reviews were excluded. Full texts of potential articles were further analysed to determine whether they met the inclusion criteria. Forty-two articles were retrieved in full and read. Thirty articles were removed as they were found to be outside the scope of the review and did not meet inclusion criteria, focusing specifically on visually impaired patients’ navigation ability with tactile substitution. A further eight studies were removed as the authors investigated SS in a cohort of healthy adults. Four studies [30, 31, 32, 33] were deemed to fit the scope of this review and therefore included to be appraised and synthesised.

References of the included articles were searched and screened for additional relevant research, and five additional studies were retrieved in full and read. These studies [34, 35, 36, 37, 38] were deemed to meet the scope of this review and therefore were included. No further relevant trials were identified from the clinicaltrials database. After inclusion, the study characteristics, research goals, and main findings with respect to balance, gait, and functional performance were extracted and summarized (see Appendix 1).

The breakdown of retrieved article is demonstrated and formatted using a PRISMA flow diagram in Figure 1 [39].

#### 3.1.2 Study characteristics

*Appendix 1* illustrates characteristics, intervention/feedback modality, SS format and placement, assessment time points, results and gaps or limitations of the included studies.

Nine RCTs were included in this SLR and MA and investigated participants with Parkinson's disease (PD) (n = 5) [32,[34], [35], [36], [37]], stroke (n = 3) [33,36,38] and vestibular disorders (n = 2) [[30], [31]]. The mean age of the samples ranged from 52 to 76 years. Sample sizes ranged from 8 to 38. Dropouts were reported in seven of the studies with similar rates between the experiment and control groups [30, 31, 33, 34, 35, 36, 38]. In total, 196 participants were recruited across Experimental and Control groups, with 182 adhering to the study until completion. Therefore, there was a 92.86% adherence rate to the intervention and controls across the included studies. Reasons given for the 14 patient dropout included non-related medical reasons, issues with transportation, death, 1 patient reported handling issues with the SS apparatus and 1 patient dropped out without giving any formal reason.

**Figure 2:**
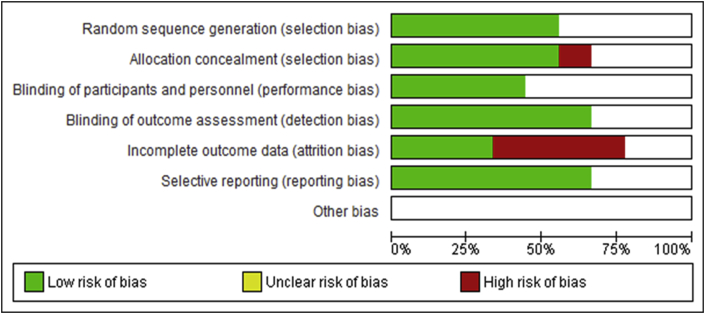
Risk of bias graph: review authors judgments about each risk of bias item presented as percentages across all included studies.

**Figure 3:**
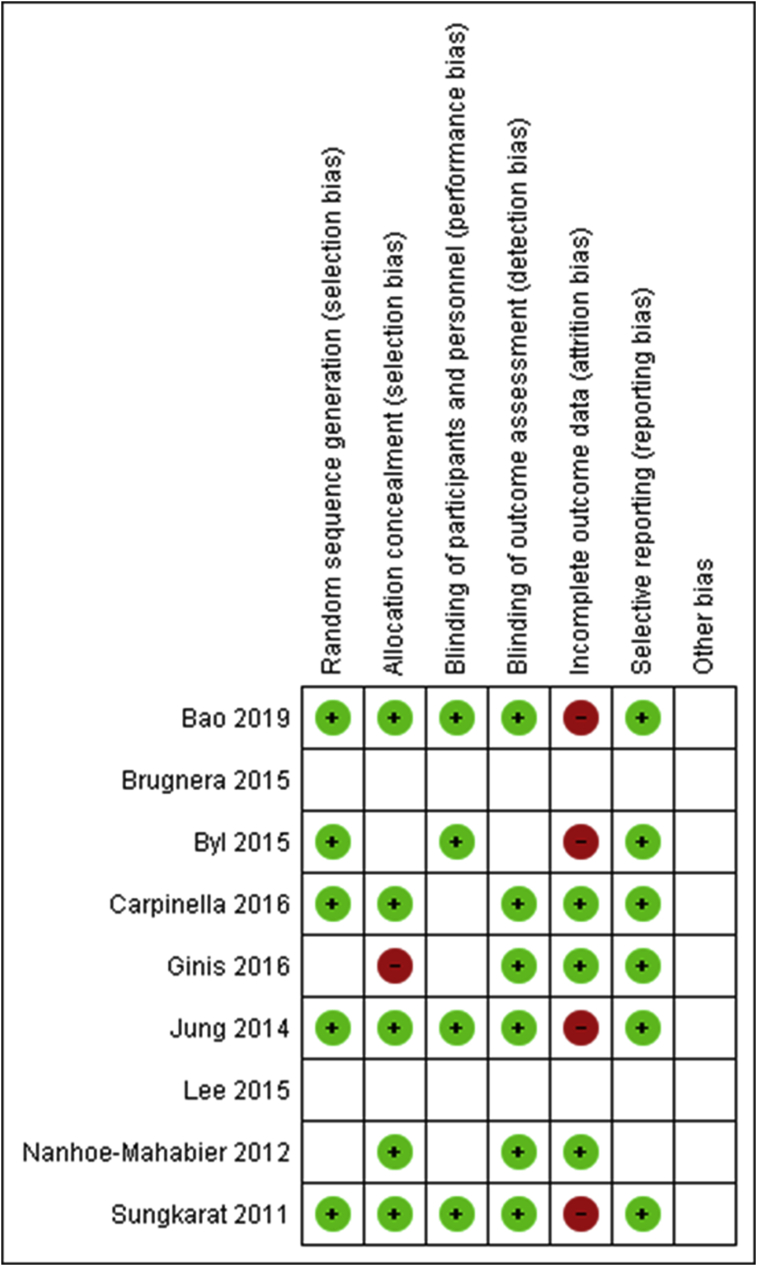
Risk of bias summary: review authors' judgements about each risk of bias item for each included study.

The mean quality score on the BMJ Framework for appraising 2-armed Randomised Controlled Trials was 15.5 (out of 20 points, range 11–18). Five of the studies were appraised as “high quality” (score 16–20) [30,33,34,36,38], three studies appraised as “moderate quality” (score 12–16) [31,35,37] and one study was appraised as “low quality” (score <12) [32]. Please see *Appendix 2* for breakdown of appraisal scores. The most frequent methodological limitations were a lack of blinding or reporting on blinding, unclear intention-to-treat analysis, and most of the interventions not being generalizable to routine care due to the SS devices used. The Cochrane Risk of Bias Assessment Tool was also applied demonstrating judgements about each risk of bias item presented as percentages across all included studies (Figure 2) and judgements about each risk of bias item for each included study (Figure 3) [26]. Four studies [30, 33, 35, 38] presented a low risk of bias. Five studies [31, 32, 34, 35, 37] presented unclear risk of bias in “blinding of participants and personnel” and four studies [30, 33, 36, 38] presented high risk of bias in “incomplete outcome data”. Rationale for each judgement was structured using the application of the BMJ Framework for appraising 2-armed Randomised Controlled Trials [25].

#### 3.1.3 Sensory Substitution paradigms used

##### 3.1.3.1 Intervention

Intervention duration ranged from 1 day to 8 weeks [30, 31, 32, 33, 34, 35, 36, 37, 38]. Intervention volume ranged from 1 to 18 training sessions. In eight studies, the control group received the same training as the intervention group, however, without SS feedback [30, 31, 33, 34, 35, 36, 37, 38]. In one study, the control group received both the same intervention and SS feedback, with the only distinguishing variable being the intervention group consisted of PD participants and the control comprised of healthy adults [32]. One study provided home-based smartphone-delivered automated feedback training [34], five were based in a clinical setting [30, 33, 35, 36, 38], whilst the remaining studies did not explicitly disclose the setting of the intervention [31, 32, 37].

According to the framework of Shumway-Cook and Woollacott [27], training paradigms used in the included RCTs varied across the framework's balance paradigms.

- Static steady-state balance training. Eight studies used static stance tasks for balance training [30, 31, 33, 34, 35, 36, 37, 38]. Each task was progressive with increasing difficulty by reducing the base of support and increasing sensorimotor demand. During the intervention, either the postural sway was measured by Inertial Measurement Units (IMUs) [30, 31, 34, 35, 37] or the weight distribution was measured by wearable plantar pressure sensors [33, 36, 38].
- Dynamic steady-state balance training. Six studies used progressively challenging gait tasks [30, 33, 35, 36, 37, 38]. One study instructed participants to walk as regular [34]. During the intervention, gait parameters were measured either by IMUs [30, 33, 34, 35, 37] or wearable plantar pressure sensors [36, 38].
- Proactive balance training. Five studies used task orientated actions for proactive balance training [30, 34, 35, 36, 38]. During the intervention, either sway was measured by IMUs [30, 34, 35] or weight distribution was measured by wearable plantar pressure sensors [36, 38].

##### 3.1.3.2 Sensory Substitution/augmentation format and placement

Seven studies used Inertial Measurement Units (IMUs), including accelerometers, gyroscopes and magnetometers, to measure thresholds for biofeedback [30, 31, 32, 34, 35, 36, 37]. IMUs are electronic devices that measure and report specific force, angular rate, and orientation of the body. The number of IMUs used in the research ranged from 6 [34] to 2 [34,37] or 1 [30-32]. One study combined IMUs and pressure sensors [36]. The final two studies used only pressure sensors [33, 38]; one used an insole plantar pressure sensor [38], whilst the other used a vertical force detecting walking cane [33] (see Appendix 1 for more detail).

IMUs measured specific segmental body kinematic data to provide feedback about centre of mass [30, 31, 32, 35, 37] or lower extremity movement [34, 35, 36]. Pressure sensors were used to detect and measure weight-bearing symmetry and variation during gait [33, 36, 38]. In all included studies, the data accumulated from IMUs and pressure sensors was translated into either visual, auditory or vibrotactile feedback, or a combination of two of the feedbacks [32, 35].

### 3.2 Feedback provided during training

Different modes of SS feedback were provided, dependent on the training paradigm used.

- Static steady-state balance training. Realtime corrective feedback about postural sway was provided either by substituting vestibular information through tactile vibrations to the lower back [30, 31], tactile vibrations via a headband [37], visual kinematic indicators of weight distribution [36], acoustic transducers [38], or a combination of acoustic transducers and visual feedback [35], when postural sway exceeded the threshold predetermined during baseline assessment.
- Dynamic steady-state balance training. Biofeedback on postural sway during gait was substituted by tactile vibrations to the lower back [30, 31] and via a headband [37]. Perception of weight symmetry was substituted by acoustic transducers [33, 38]. Perception of gait variables were substituted visually with kinematic information projected onto a monitor [36]. Perception of postural sway and lower limb movements during gait were substituted by a combination of acoustic transducers and visual kinematic information projected onto a monitor [35]. Visual and auditory kinematic biofeedback provided both positive and negative biofeedback in two studies [35, 36]. One study provided visual kinematic biofeedback on gait variables, simultaneously indicating variables being achieved and providing constructive instruction [36], whilst the other provided both visual and auditory kinematic feedback via a computerised avatar which indicated ability to maintain balance within a set limit of stability [35].
- Proactive balance training. Studies with interventions investigating task orientated actions also involved vibrotactile, visual and auditory feedback. Vibrotactile biofeedback was provided via vibrations to the lower back [30, 31]. As discussed previously, perception of gait variables, postural sway and lower limb movements during gait were substituted both visually [36] and a combination of visual and auditory biofeedback [35], with both positive and negative biofeedback provided [35, 36]. Auditory feedback was provided via diverse sources [34, 35, 38]. A combined auditory and visual kinematic feedback was provided as discussed previously [35]. Positive auditory verbal feedback via an app was given when gait remained within a set therapeutic threshold, with corrective auditory verbal feedback given when gait parameters fell outside thresholds [34]. An audio signal was emitted to indicate when/if weight-bearing load through an insole for a paretic leg exceeded a set threshold [38].

### 3.3 Meta-analysis

Due to the diversity of the outcome measures used in the included studies, each MA incorporates a combination of training paradigms, where each outcome was analysed under the training paradigm it effected.

For all training effect analysis, a SMD was used to calculate the intervention effect size as studies used varying measures to evaluate outcomes.

#### 3.3.1 Effects on Static Steady-State Balance measures

**Figure 4:**
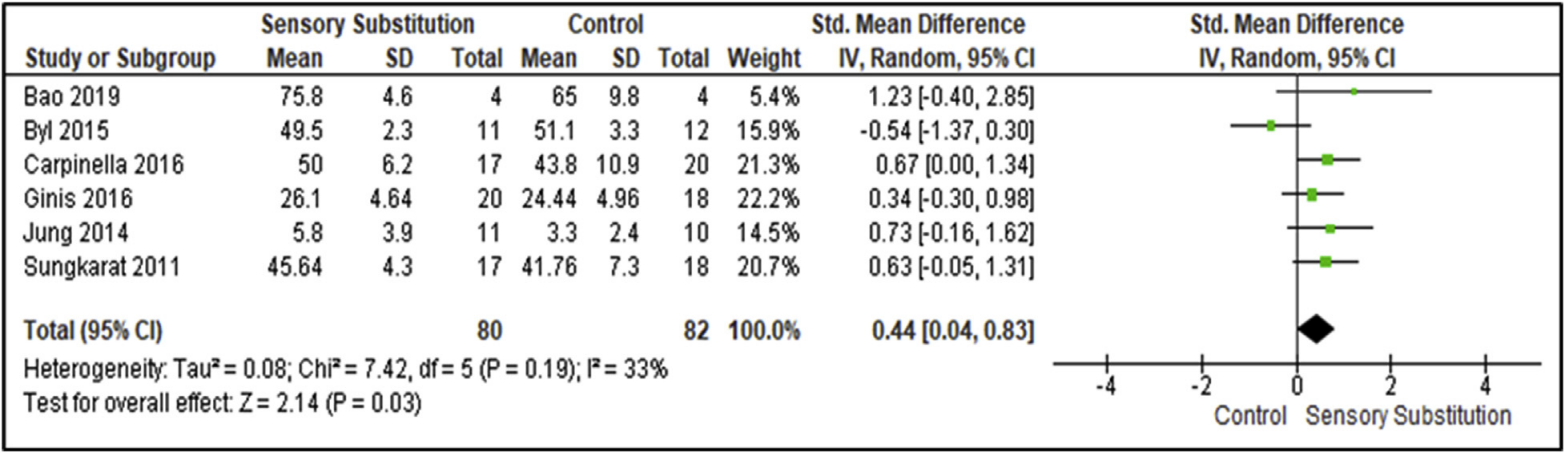
Training effects on Balance; SS vs Control.

Six studies compared training effects on static postural sway and balance measures between Experiment and Controls [30, 33, 34, 35, 36, 38] (Figure 4). These studies included 80 participants in the Experimental groups and 82 in the Control groups. Outcome measures used in the studies to assess this training paradigm were the Sensory Organisation Test (SOT), Mini-Balance Evaluation Systems Test (Mini-BESTest) and Berg Balance Scale (BBS). There was a non-significant level of heterogeneity between studies (I^2^ = 33%; P = 0.19) [28]. A MA of these studies revealed a significant overall effect of SS training when compared with controls (P = 0.03; SMD = 0.44; 95% CI: 0.04, 0.83; random-effects model).

**Figure 5:**
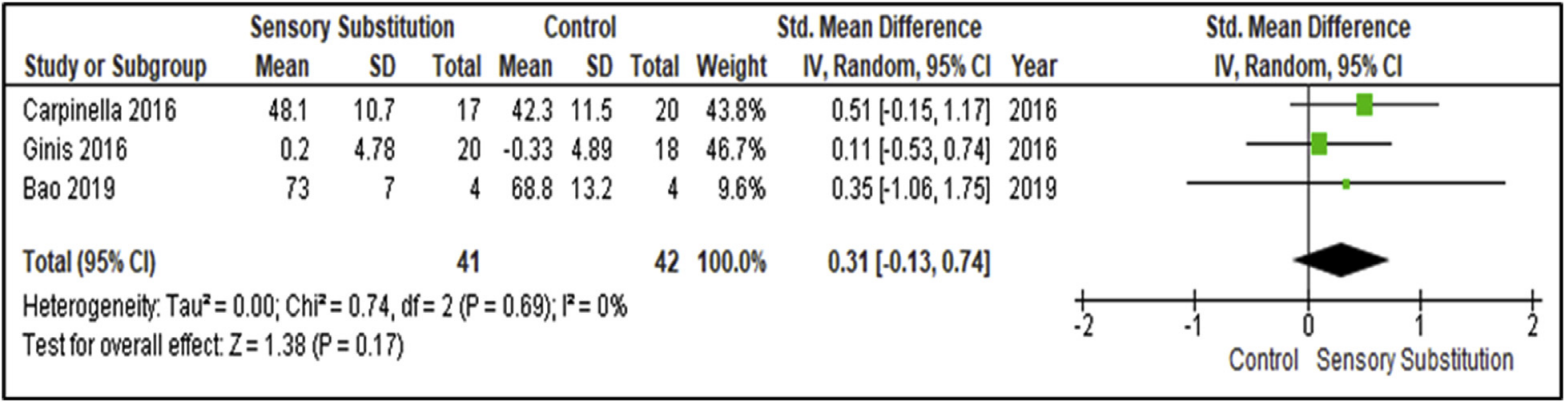
Training effects on Balance Retention; SS vs Control.

The effects above were measured immediately after the intervention. Three studies performed follow-up measurements after 1 month or 6 months and recorded outcomes to access retention of effects [30, 34, 35] (Figure 5). These studies included 41 participants in the Experimental groups and 42 in the Control groups. Outcome measures once again used in the studies were the SOT, Mini-BESTest and BBS. There was a non-significant level of heterogeneity between studies (I^2^ = 0%; P = 0.69) [28]. A MA of the studies revealed a non-significant overall retention effect of SS training when compared with controls (P = 0.17; SMD = 0.31; 95% CI: -0.13, 0.74; random-effects model).

#### 3.3.2 Effects on Dynamic Steady-State balance measures

**Figure 6:**
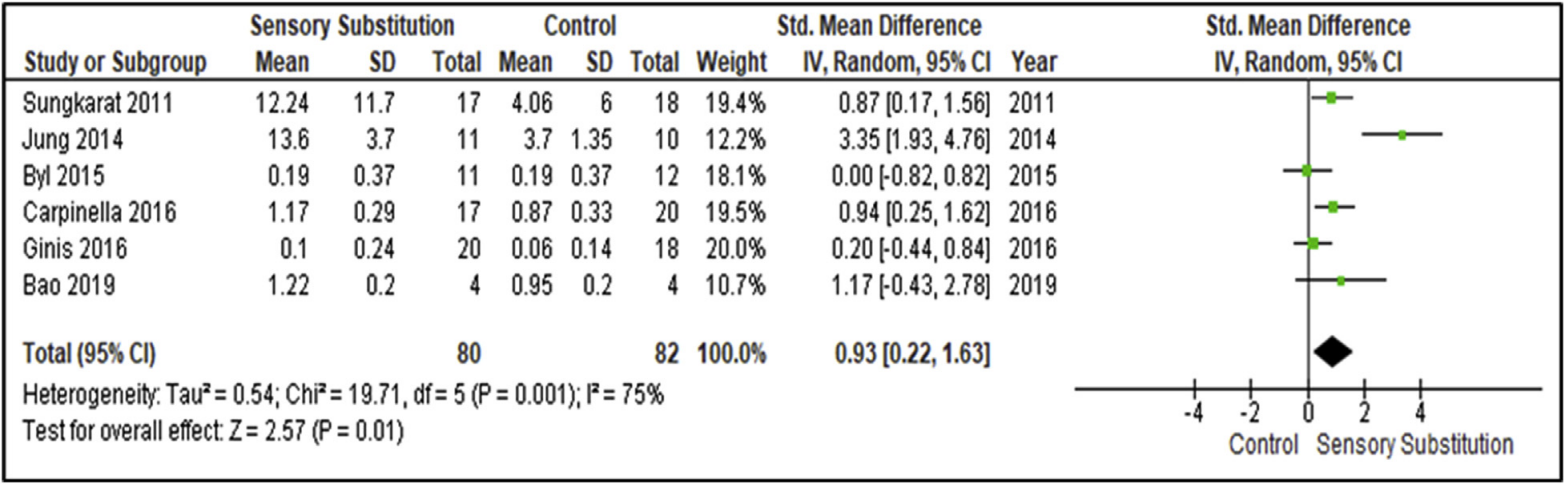
Training effects on Gait speed; SS vs Control.

Six studies evaluated training effects on gait performances by reporting on different gait parameters [30, 33, 34, 35, 36, 38] (Figure 6). These studies included 80 participants in the Experimental groups and 82 in the Control groups. The most consistent gait parameter/outcome measure across the studies was habitual gait speed. There was considerable heterogeneity between studies for this outcome (I^2^ = 75%; P = 0.001) [28]. When analysing sources of heterogeneity, the study by Jung and colleagues was found to represent an outlier, with a standardised effect size of 3.35 compared to the overall standardised effect size of 0.93 [33]. When removed from the analysis, homogeneity between the studies is met (I^2^ = 28%; P = 0.24) [28]. The study is included in the meta-analysis for a universal analysis. A MA of these studies revealed a significant overall effect of SS training when compared with controls (P = 0.01; SMD = 0.93; 95% CI: 0.22, 1.63; random-effects model).

**Figure 7:**
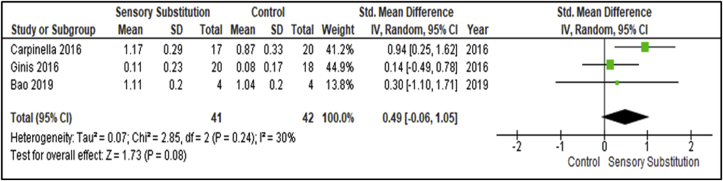
Training effects on Gait speed Retention; SS vs Control.

The effects above were measured immediately after the intervention. Three studies performed follow-up measurements after 1 month or 6 months and recorded outcomes to access retention of effects [30, 34, 35] (Figure 7). These studies included 41 participants in the Experimental groups and 42 in the Control groups. Once more, the most consistent gait parameter/outcome measure across the studies was habitual gait speed. Parameters of homogeneity were met between studies (I^2^ = 30%; P = 0.24) [28]. A MA of the studies revealed a non-significant overall retention effect of SS training when compared with controls (P = 0.08; SMD = 0.49; 95% CI: -0.06, 1.05; random-effects model).

#### 3.3.3 Effects on proactive balance measures

**Figure 8:**
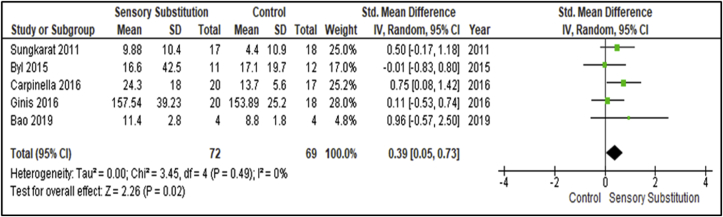
Training effects on Proactive Balance; SS vs Control.

Five studies measured the effects on proactive balance function [30, 34, 35, 36, 38] (Figure 8). These studies included 72 participants in the Experimental groups and 69 in the Control groups. Outcome measures used in the studies to assess this training paradigm were the Timed Up-and-go (TUG), 2 Minute Walk Test (2MWT) and 6 Minute Walk Test (6MWT). There was no evidence of heterogeneity between studies (I^2^ = 0%; P = 0.49) [28]. A MA of these studies revealed a significant overall effect of SS training when compared with controls (P = 0.02; SMD = 0.39; 95% CI: 0.05, 0.73; random-effects model).

#### 3.3.4 Effects on other outcomes

**Figure 9:**
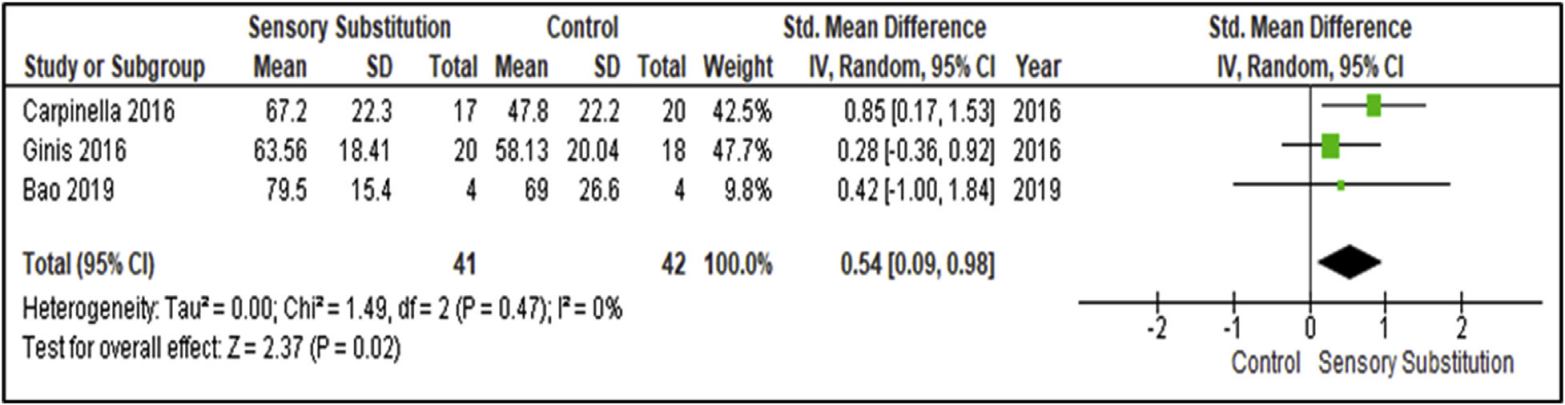
Training effects on Self-assessment; SS vs Control.

Three studies compared training effects on self-assessment between the Experiment and Control groups [30, 34, 35] (Figure 9). These studies included 41 participants in the Experimental groups and 42 in the Control groups. Outcome measures used in the studies to assess this outcome were the Activities-Specific Balance Confidence (ABC) Scale and the Short Form 36 Health Survey Questionnaire - Mental Health (SF-36 MH). There was no evidence of heterogeneity between studies (I^2^ = 0%; P = 0.47) [28]. A MA of these studies revealed a significant overall effect of SS training when compared with controls (P = 0.02; SMD = 0.54; 95% CI: 0.09, 0.98; random-effects model).

**Figure 10:**
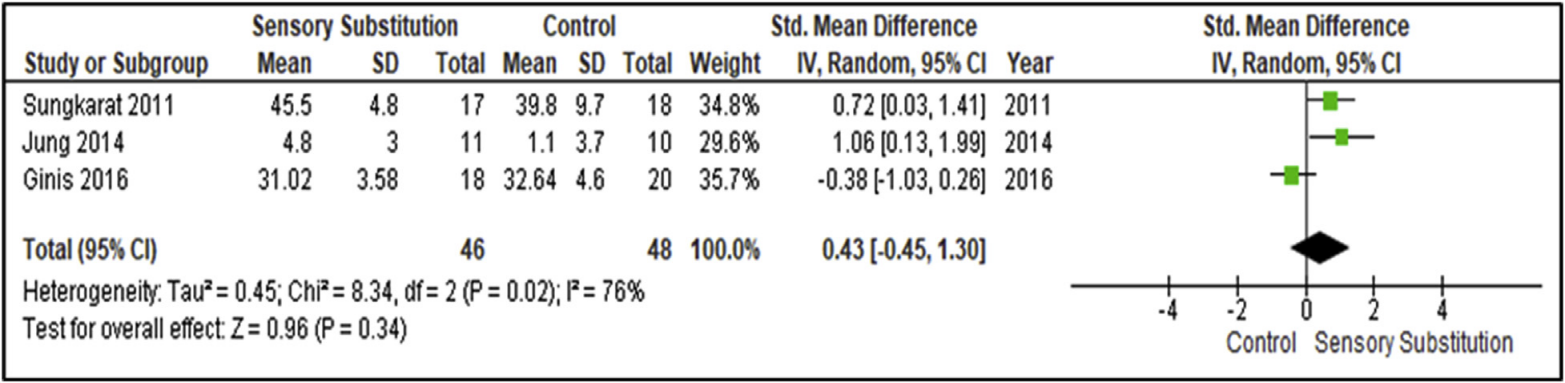
Training effects on overall single limb support time; SS vs Control.

Additionally, three studies measured the duration participants could support themselves on a single lower limb between Experiment and Control groups (33; 34; 38) (Figure 10). The cohort analysed included stroke (n = 56) and PD participants (n = 38), with stroke participants’ single limb support duration measured for their paretic side. These studies included 46 participants in the Experimental groups and 48 in the Control groups. There was considerable heterogeneity between studies for this outcome (I^2^ = 76%; P = 0.02) [28]. A MA of these studies revealed a non-significant overall effect of SS training when compared with controls (P = 0.43; SMD = 0.43, 95% CI: -0.45, 1.30; random-effects model).

**Figure 11:**
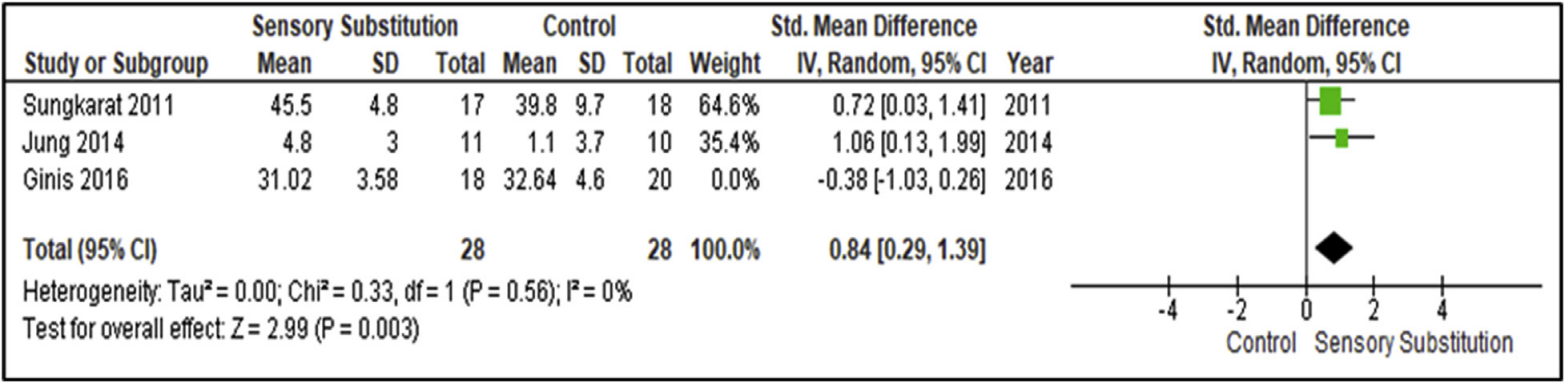
Training effects on single limb support time sub-analysis; SS vs Control.

When sub-analysing sources of heterogeneity, the study by Ginis and colleagues was found to represent an outlier for this outcome, with a standardised effect size of -0.38 compared to the overall standardised effect size of 0.43 [34]. Interestingly this study included the PD cohort, and when removed from the analysis, thus solely analysing stroke participants, homogeneity between the studies is met (I^2^ = 0%; P = 0.56) [28], and also a MA revealed a significant overall effect of SS training when compared with controls (P = 0.003; SMD = 0.84, 95% CI: 0.29, 1.39; random-effects model) (Figure 11).

## 4 Discussion

### 4.1 Main findings

The aim of this SLR and MA was to examine and estimate the effect of balance, gait and functional training supplemented by SS in neurological patient populations. This MA showed significant effects of the intervention in improving Static steady-state, Dynamic Steady-State and Proactive balance parameters compared to controls. The MA also showed significant effects of SS supplemented training in improving neurological patients' self-efficacy and stroke patients’ capacity to weight bear through their paretic lower limb. In contrast, the MA revealed non-significant effects of the intervention in retention/long term effects of improved balance and gait parameters after the trial concluded.

A total of nine studies were retrieved for inclusion in this SLR and MA. Five studies [33, 34, 35, 36, 38] clearly presented the data necessary to conduct the MA, whilst four [30, 31, 32, 37] lacked accessible data. Correspondence for these four studies where gathered and contacted, with two authors [30, 32] responding with the additional necessary data. One author [30] provided Mean and Standard Error of the Mean (SEM) data, from which the lead author calculated the Standard Deviation (SD) for statistical analysis. No response was received from the remaining authors [31, 37], which therefore resulted in exclusion from the MA due to missing data.

IMUs were the data gathering instruments most frequently used in the studies included, but placement varied across the studies. IMUs were mounted on the lower back via a belt device [30, 31, 32, 37], or placed on shoes for gait variable feedback [34, 36], or placement being spread out across the upper trunk, lower trunk and lower limbs to gather large surface area kinematic data for biofeedback [35]. Other than IMUs, pressure sensors incorporated into an insole and walking cane were used [33, 38]. These varying sensor types and placements limited the overall comparability of included studies, with the volume examining each too low to perform a sub-analysis according to sensor type, placement or type of biofeedback.

Regarding the volume of balance training recommended for neurological patient populations, no SLR or specific dosage guidelines were found. A secondary analysis of the Locomotor Experience Applied Post-Stroke (LEAPS) RCT by Rose and colleagues [40] evaluated the dose-response relationship between locomotor training and strength and balance exercises on gait recovery poststroke. According to the findings, for both interventions, a training period of 8–12 weeks, with a frequency of 3 times per week, a total number of 24–30 training sessions and of 90-minute duration induces the largest effect on gait performance [40]. In contrast, this SLR revealed none of the included SS supplemented training interventions achieved this proposed optimal dosage [40]. The training period of the included interventions were shorter (1 day–8 weeks), with only one study intervention reaching 8 weeks [36]. Intervention volume was also much lower, with total training sessions ranging from 1 to 18 sessions compared to the 24–30 recommended. Intervention duration was again much lower, with the longest intervention duration being 45 min [35] compared to the 90 min recommended [40]. Despite not achieving recommended training dosage, SS supplemented training was found to have significant effects on each balance paradigm examined in this MA. However, this low training dosage may explain the limited effects found for other outcomes, i.e. the retention of balance and gait effects.

An important aspect of motor learning is the differentiation between positive and corrective feedback. While positive feedback fosters motivation, corrective feedback is an essential element during learning processes [41]. Whether positive or corrective feedback is more effective for recovery through SS supplemented training is unclear as no guidance is available. RCTs included in this SLR and MA provided either corrective feedback [30, 31, 32, 33, 37, 38] or a combination of this with positive feedback [34, 35, 36], no study solely included positive feedback. An example of corrective feedback in this SLR and MA is the study from Jung and colleagues [33], in which an auditory sound was emitted when participants exceeded a set weight-bearing threshold through a vertical force detecting walking cane. An example of combined feedback comes from the study by Byl et al. [36], in which visual kinematic feedback was provided regarding gait variables, simultaneously providing positive reinforcement with green coloured messages including “you are doing perfectly!” and red coloured constructive instruction including “step further on the LEFT!” [36]. The effects between positive and corrective feedback on outcome measures were not compared in any of the included research.

### 4.2 Effects on Static Steady-State Balance measures

This MA revealed a significant overall effect of SS supplemented training for Static Steady-State Balance measures (P = 0.03; SMD = 0.44; 95% CI: 0.04, 0.83; random-effects model). Whilst these findings are statistically significant (p = 0.03), the clinical effect of the intervention compared to controls is regarded as Small [29]. The statistical significance finding is supported by a SLR and MA by Gordt et al. [18] who analysed the effects of SS supplemented training on balance, gait, and functional performance in healthy and diverse patient populations.

Balance disturbance is a common, difficult to treat cause of significant morbidity and mortality in neurological patient populations. Much of the long-term disability of PD is reported to be related to symptoms of balance and gait disorders [42], whilst recovery of balance and gait is one of the main aims and motivators in stroke rehabilitation [8]. These findings appear to correlate with research reporting that functional ability to conduct personal care and Activities of Daily Living (ADLs) are reported below only ‘family’ in importance for life goals of people with neurological disorders [43]. Therefore, interventions such as SS which are suggested to improve balance and facilitate important life goals are vital to neurological patient populations, especially those with progressive disorders, who are reported to progressively consider less life goals important over time [44].

Regarding effect retention, although the number of studies which included follow-up assessments was low (n = 3), a MA revealed a statistically non-significant overall retention effect of SS supplemented training on Static Steady-State Balance measures when compared with controls (P = 0.17; SMD = 0.31; 95% CI: -0.13, 0.74; random-effects model). Whilst also displaying improvements compared to controls, the clinical effect of retention for this paradigm is once more Small [29]. As discussed previously, when compared to guidelines, none of the SS supplemented training studies included in this review met proposed optimal training dosage [40]. This may be rationalised by symptoms related to neurological disorders, such as fatigue. It is important to note that although symptom-related adherence issues are not objectively examined and cannot be assumed, evidence suggest it warrants consideration. The prevalence of fatigue is elevated in many neurologic illnesses beyond what would be expected solely based on age and disability, including MS [45], PD [46] and stroke [47]. Fatigue is associated with decreased quality of life and increased disability in these conditions, even when controlling for other symptoms, such as depression [48, 49, 50]. It may be difficult to achieve high levels of adherence to lengthy training programs and follow-up assessments amongst participant cohorts presenting with these sorts of symptoms. In order to address these factors, home-based training could be considered for delivery of future SS supplemented training. Findings from a SLR suggest a significant effect in favour of home-based over clinic-based rehabilitation, in terms of function, satisfaction and cost benefit for people living with stroke [51].

### 4.3 Effects on Dynamic Steady-State balance measures

This MA revealed a very significant overall effect of SS training for Dynamic Steady-State Balance measures (P = 0.01; SMD = 0.93; 95% CI: 0.22, 1.63; random-effects model). The intervention was found to have significant effects both statistically (P = 0.01) and clinically (SMD = 0.93), with a Large clinical effect [29]. This finding is in contrast with the SLR and MA by Gordt et al. [18], who reported no significant effects for this paradigm. As Gordt and colleagues [18] included healthy patient populations and no population sub-analysis, future research could examine which population benefit most and least from SS supplemented training. Gordt and colleagues [18] also discuss that their findings suggest patients with a specific neurological deficit would benefit particularly from SS supplemented training [18].

Habitual gait speed was the most frequently assessed gait parameter in the included research for this SLR and MA and the comparative review [18]. Interventions to increase gait speed warrants consideration as part of rehabilitation as evidence suggests gait speed is a key predictor of community mobility after neurological disruption [52]. Gait speed was found to be more significantly related to community mobility than gait tolerance/endurance [52].

Although the number of studies which included follow-up assessments was low (n = 3), a MA revealed a statistically non-significant and Small clinical overall retention effect of SS supplemented training on Dynamic Steady-State Balance measures when compared with controls (P = 0.08; SMD = 0.49; 95% CI: -0.06, 1.05; random-effects model) [29]. As discussed previously, a lack of interventions meeting proposed optimal training dosage may influence retention of effects. As reported, although not objectively examined and cannot be assumed, symptoms associated with these populations may make it difficult to achieve high levels of adherence to lengthy optimal dosage training programs and follow-up assessments.

### 4.4 Effects on proactive balance measures

This MA revealed a significant overall effect of SS supplemented training for Proactive Balance measures (P = 0.02; SMD = 0.39; 95% CI: 0.05, 0.73; random-effects model). The intervention was found to have statistically significant effects (P = 0.02) but a Small clinical effect (SMD = 0.39) compared to controls [29]. As with Dynamic Steady-State Balance measures, this finding is in contrast with the results of Gordt et al. [18], who reported no significant effects for this training paradigm. Once more, as the authors [18] included no sub-analysis, future research could examine which population benefit most and least from SS supplemented training related to this paradigm.

The outcome measures included in this MA are used to assess falls risk. Falls represent one of the most common complications following a stroke [53] and a significant cause of disability, lost independence, and reduced quality of life in people with PD [54]. The findings of this MA suggest SS supplemented training reduced risk of falls for these populations. This warrants cautious consideration for the training to be an addition to falls prevention programmes for neurological rehabilitation until a larger more demographically dense evidence base is gathered.

### 4.5 Effects on other outcome measures

This MA revealed a significant overall effect of SS supplemented training for self-assessment (P = 0.02; SMD = 0.54; 95% CI: 0.09, 0.98; random-effects model) and paretic side single lower limb support time (P = 0.003; SMD = 0.84, 95% CI: 0.29, 1.39; random-effects model). The intervention was found to have statistically significant effects (P = 0.02) and Medium clinical effect (SMD = 0.54) on self-assessment and statistically significant effects (P = 0.003) and Large clinical effect (SMD = 0.84) on paretic side single lower limb support time [29]. These findings included analysis of participant cohorts with Unilateral Vestibular Disorders (UVD) [30] and PD [34, 35] for self-assessment measures, and stroke survivors for paretic side single lower limb support time respectively [33, 38]. No comparative outcome measures were assessed by Gordt and colleagues [18] for these analyses.

Recent evidence has found an association between self-efficacy and objective walking performance in MS, with higher self-efficacy found to be a strong predictor of increased gait speed and tolerance [55]. Findings from a recent review [56] found trunk and lower limb motor control appear to be the greatest predictors of gait recovery post-stroke, with research reporting that recovery of gait is one of the main aims and motivators in stroke rehabilitation [8, 56]. As this MA suggests SS supplemented training significantly improves both self-efficacy and lower limb motor control, there should be cautious consideration for the training to be an addition to neurological rehabilitation, although, once more, a larger more demographically dense evidence base is required.

### 4.6 Methodological consideration

The use solely of the Intervention section of the PICO formatted search strategy presents a possible foundational constraint of this review due to the potentially relevant research missed in the database search. As no MESH for SS devices were found, a narrow search string using only Intervention keywords was used, in order to produce the most relevant research. This was conducted in order to preserve a concise scope as per the PRISMA-P checklist [19], and as used in previous literature [20]. As over half of the articles (n = 5) included in this SLR and MA were accumulated via an exploration of references of the articles yielded through the search strategy, it is clear that development of MESH for SS is vital to guide future literature to distinguish SS supplemented training interventions from other rehabilitative devices, including non-substitution stimulation devices.

## 5 Conclusion

To our knowledge, this is the first SLR and MA of RCTs examining SS supplemented balance, gait and functional training, exclusively examining neurological patient populations. This review updates and adds to understanding produced by a SLR and MA by Gordt et al. [18], who analysed SS supplemented balance, gait and functional training in healthy adults and varied patient populations. In summary, our results indicate that there is evidence in favour of a global positive effect of SS supplemented training in improving Static Steady-State, Dynamic Steady-State and Proactive balance measures, as well as measures of self-assessment and functionality variables. Findings of this MA suggest the most significant statistical and clinical effects of the intervention are in favour of improving Dynamic Steady-State balance and the ability of stroke survivors to support bodyweight independently on the paretic side lower limb. No study has trained and assessed Reactive balance measures. Future research might include this balance paradigm, as it has been reported to be important in falls prevention [57].

Both positive and negative effects of SS supplemented training were found in this review, dependent on the outcome measure being examined. Methodological weaknesses in the included RCTs were also found, the most frequent being a lack of blinding or reporting on blinding, unclear intention-to-treat analysis, and most of the interventions not being generalizable to routine care. Also, no included study intervention was found to meet suggested optimal training dosage for neurological patient populations.

Considering only nine studies across three clinical populations, with small sample sizes and mixed quality across the research were found, some caution should be taking when interpreting the effects SS interventions in neurological patient populations until a larger more demographically dense evidence base is available.

Clear comprehensive training dosage guidelines for neurological patient populations should be developed to provide a foundational template for future trials to follow. Future research should consider optimal dosage of training and follow-up assessments when designing trials. Home-based training should be considered for future research settings to accommodate high training dosage. It is important for future research to consider variables such as specific patient population, sensor type, and training modalities to conduct subgroup analysis and identify the most effective variables of SS supplemented training. Finally, it is important that future reviews on the topic of SS supplemented training are of high methodological quality and are regularly published to keep best evidence up to date.

## 6 Declarations

### Author contribution statement

All authors listed have significantly contributed to the development and the writing of this article.

### Funding statement

This work was supported by Institute of Technology Sligo Research Masters Scholarship Fee Waiver.

### Data availability statement

Data included in article/supplementary material/referenced in article.

### Declaration of interests statement

The authors declare no conflict of interest.

### Additional information

No additional information is available for this paper.

## Appendix A Supplementary data

Summary of included articles Summary of Included Article Characteristics Summary of included articles

Critical Appraisal of included articles Application of BMJ Framework for appraising 2-armed Randomised Controlled Trials Critical Appraisal of included articles
