# Supplementary material for: Effects of sensory substituted functional training on balance, gait, and functional performance in neurological patient populations: A systematic review and meta-analysis
Source: Heliyon. 2021 Sep 17;7(9):e08007. doi: 10.1016/j.heliyon.2021.e08007 (PMC8473554; doi:10.1016/j.heliyon.2021.e08007)
Supplement: Summary of included articles — Summary of Included Article Characteristics [file mmc1.docx]

| **Study**  **Criteria** | **Bao et al. 2019** | **Ginis et al. 2016** | **Carpinella et al. 2016** | **Brugnera et al. 2015** | **Lee et al. 2015** | **Byl et al. 2015** | **Jung et al. 2014** | **Nanhoe-Mahabier et al. 2012** | **Sungkarat et al. 2011** |
| --- | --- | --- | --- | --- | --- | --- | --- | --- | --- |
| Is the included population explicitly described? | ✓ | x | ✓ | ✓ | ✓ | ✓ | ✓ | ✓ | ✓ |
| Are the intervention and control explicitly described? | ✓ | ✓ | ✓ | ✓ | ✓ | ✓ | ✓ | ✓ | ✓ |
| Is the setting explicitly described? | ✓ | ✓ | ✓ | x | x | ✓ | ✓ | x | ✓ |
| Are the outcomes explicitly described? | ✓ | ✓ | ✓ | ✓ | ✓ | ✓ | ✓ | ✓ | ✓ |
| Are the Outcome measures Valid? | ✓ | ✓ | ✓ | ✓ | ✓ | ✓ | ✓ | ✓ | ✓ |
| Is the study adequately randomised? | ✓ | ✓ | ✓ | x | x | ✓ | ✓ | x | ✓ |
| Is the randomisation method described? | ✓ | ✓ | ✓ | x | ✓ | ✓ | ✓ | x | ✓ |
| Is the level of blinding reported? | ✓ | ✓ | ✓ | x | x | x | ✓ | x | ✓ |
| Could the level of blinding affect the interpretation of the results? | ✓ | x | x | x | x | x | ✓ | x | ✓ |
| Are both groups treated in exactly the same way apart from the intervention? | ✓ | ✓ | ✓ | ✓ | ✓ | ✓ | ✓ | ✓ | ✓ |
| Are the treatment and control groups similar at baseline? | ✓ | ✓ | x | ✓ | x | ✓ | ✓ | ✓ | ✓ |
| Are any baseline differences explained and/or accounted for at analysis? | ✓ | ✓ | x | ✓ | ✓ | ✓ | ✓ | ✓ | ✓ |
| Are all randomised people accounted for at the end of the trial? | ✓ | ✓ | ✓ | ✓ | x | ✓ | ✓ | ✓ | ✓ |
| Does the trial present an intention-to treat analysis? | x | ✓ | x | x | x | x | x | ✓ | x |
| Are final results based on more than 80% of people randomised? | ✓ | ✓ | ✓ | ✓ | x | ✓ | ✓ | ✓ | ✓ |
| Are all clinically relevant outcomes reported? | ✓ | ✓ | ✓ | ✓ | ✓ | ✓ | ✓ | ✓ | ✓ |
| Does the trial report a direct statistical analysis between groups? | ✓ | ✓ | ✓ | x | ✓ | ✓ | ✓ | ✓ | ✓ |
| Does the trial report appropriate statistical tests? | ✓ | ✓ | ✓ | ✓ | ✓ | ✓ | ✓ | ✓ | ✓ |
| Does the trial report on clinical relevance/ of results? | ✓ | ✓ | ✓ | ✓ | ✓ | ✓ | ✓ | ✓ | ✓ |
| Are the setting and intervention generalizable to routine care? | x | ✓ | x | x | x | x | x | x | x |
| **Criteria met** | **18/20** | **18/20** | **15/20** | **12/20** | **11/20** | **16/20** | **18/20** | **14/20** | **18/20** |

✓ = criterion is evidenced in article;

x = criterion is not evidenced or could not be determined in article
