# Supplementary material for: Effects of sensory substituted functional training on balance, gait, and functional performance in neurological patient populations: A systematic review and meta-analysis
Source: Heliyon. 2021 Sep 17;7(9):e08007. doi: 10.1016/j.heliyon.2021.e08007 (PMC8473554; doi:10.1016/j.heliyon.2021.e08007)
Supplement: Critical Appraisal of included articles — Application of BMJ Framework for appraising 2-armed Randomised Controlled Trials [file mmc2.docx]

| **Author (Country)** | **Topic / Focus** | **Sample** | **Intervention / feedback modality** | **Sensory Substitution / Augmentation format and placement** | **Assessment time points** | **Results** | **Gaps / Limitations** |
| --- | --- | --- | --- | --- | --- | --- | --- |
| Bao et al. 2019  (United States) | To investigate the effects of incorporating vibrotactile Sensory  Augmentation (SA) on balance performance among people with unilateral vestibular disorders (UVD). | n = 8  Experiment group (EG): n = 4  68.1±7.5 years  Male/Female:  1:3  Control group (CG):  n = 4  63.1±11.3 years  Male/Female:  1/3 | 6 weeks of  vibrotactile feedback  EG:  Balance and Gait exercises with SA  CG:  Balance and Gait exercises without SA | 1 x Inertial Measurement Unit (IMU):  Belt device;  Circumference of trunk at L4/L5 level. | Baseline  Mid-intervention    1-week post-intervention  1-month post intervention  6-months post intervention | EG CG  Balance:  Activities-specific Balance  Confidence Scale (ABC): ↑* ↑  Postural stability: ↑* ↑  Sensory Organization Test  (SOT): ↑ ↑  Mini Balance Evaluations  Systems Test  (Mini-BESTest): ↑ ↑  Gait:  Gait Speed Test: ↑ ↑  Dynamic Gait Index (DGI): ↑ ↑  Functional Gait Assessment  (FGA): ↑ ↑ | Small sample size  Vestibular Rehabilitation Therapy (VRT) prior to participation unknown |
| Ginis et al. 2016  (Belgium / Israel) | To investigate  feasibility and  effects of  real-time auditory corrective feedback and  gait training on  gait, balance  and quality of  life in Parkinson’s Disease (PD) | n = 38  EG: n = 20  Mean age: not specified  Gender: not specified  CG: *n* = 18  Mean age: not specified  Gender: not specified | 6 weeks of auditory feedback  EG:  Gait training with audio biofeedback-  gait-app  CG: Gait training without audio biofeedback-  gait-app | 2 x IMU’s + smartphone:  IMUS’s attached to shoes (when using the general gait app) and above ankles when using the FOG (Freezing of Gait)-cue app. | Baseline  Immediately  after intervention  4 weeks after  intervention | EG CG  Gait:  Gait speed: ↑ ↑  Stride length: ↑ ↑  Double support time: ↑ ↑  Balance:  MiniBESTest: ↑* ↑  Four Square Step Test: ↑ ↑  FES-I: - -  Physical capacity  2-minute walk Test (2MWT): ↑ ↑  Quality of Life:  SF-36: ↑* ↓ | Lack of assessor blinding for group allocation  Standard physiotherapy care delivered alongside intervention |
| Carpinella et al. 2016  (Italy) | To investigate the feasibility and efficacy of a novel system (Gamepad) for biofeedback for balance and gait rehabilitation in PD. | n = 37  EG: n = 17  73±7.1 years  Male/Female:  14/3  CG: n = 20  75.6±8.2 years  Male/Female:  9/11 | 6 weeks of  Visual/auditory feedback  EG:  Balance and Gait exercises with biofeedback  CG: Balance and Gait exercises without biofeedback | 6 x IMU’s:  Upper trunk; Lower trunk;  2 x IMU’s per lower limb | Baseline  Immediately  after intervention  1 month after  intervention | EG CG  Balance:  Berg Balance Scale  (BBS): ↑* ↑  ABC: - -  Postural stability: ↑* ↑  Gait:  10-metre walk test  (10MWT): ↑ ↑  Timed Up and Go (TUG): - - | Between group differences at baseline  Unbalanced group characteristics  ? significant placebo effect as CG wore no IMU’s |
| Brugnera et al. 2015  (Brazil) | To investigate the effectiveness of vibrotactile biofeedback as a sensory substitution (SS) of the vestibular system in patients with vestibular disorders. | n = 13  EG: n= 7  71±11 years  Male/Female:  6:1  CG: n = 6  71.8±16 years  Male/Female:  3/3 | 10 days of  vibrotactile feedback  EG:  Balance and Gait exercises with biofeedback  CG:  Balance and Gait exercises without biofeedback | 1 x IMU:  Belt device;  Level of device “coupled to patient’s waist” | Baseline  Immediately  after intervention | EG CG  Balance:  SOT: ↑ -  Self-assessment:  ABC: ↑ -  Dizziness Handicap Inventory  (DHI): ↑ ↑ | Small sample size  Unbalanced group characteristics  No follow-up retention assessment |
| Lee et al. 2015  (United States) | To investigate the effects of guidance modalities during common dynamic weight-shifting exercises used in  PD. | n = 20  EG: n = 11  70±8.1 years  Male/Female:  9/2  CG: n = 9  67.8±6.6 years  Male/Female:  1/7 | 1 session of Visual and/or vibrotactile feedback  EG:  Balance exercises with feedback in PD participants.  CG:  Balance exercises with feedback in healthy participants. | 1 x IMU:  Belt device;  Circumference of trunk at L5/S1 level.  Tactors placed anteriorly, posteriorly and bilaterally at L4/L5 level. | Baseline  Immediately  after intervention | EG CG  Balance:  SOT: - -  Limits of stability  (LOS): ↑ ↑  Feedback modality:  Visual: ↑ ↑  Vibrotactile: ↑ ↑  Visual & vibrotactile: ↑* ↑* | Non- randomised  Unbalanced group characteristics  Very brief intervention  No follow-up retention assessment |
| Byl et al. 2015  (United States) | To investigate the effectiveness of gait training with and without visual biofeedback to improve mobility, balance, strength and flexibility in stroke and PD. | n = 23  EG: n = 11  67.3±4.3 years  Male/Female:  5/6  Stroke/PD:  4/7  CG: n = 12  65.4±4.2 years  Male/Female:  6/6  Stroke/PD:  7/5 | 6-8 weeks of visual feedback  EG:  Transfer, gait, strength and flexibility training with biofeedback  CG:  Transfer, gait, strength and flexibility training without biofeedback | 4 x IMU’s + 8 sensors:  2 x Smart shoes; sensors placed at heel, forefoot (medial and lateral) and great toe  2 x Thigh IMU’s; sensors placed proximally and distally to knee in bilateral lower limbs | Baseline  Immediately  after intervention | EG CG  Balance:  BBS: ↑ ↑  Gait:  DGI: ↑ ↑  Tinetti Gait assessment: ↑ ↑  TUG: - -  Gait speed: ↑ ↑  Step length: ↑ ↑  6-min walk: ↑ ↑  Other measures:  Strength: ↑ ↑  Range of motion (ROM): ↑ ↑ | No follow-up retention assessment  Small sample size within diagnostic group  Smart shoes not fitted well to participants |
| Jung et al. 2014  (Republic of Korea) | The investigate the effect of gait training with an augmented pressure sensor cane for increasing muscle activation of the affected limb and improving gait in patients with stroke. | n = 21  EG: n = 11  56.4±11 years  Male/Female:  7/4  CG: n = 10  56.3±17 years  Male/Female:  7/3 | 4 weeks of  auditory feedback  EG:  Gait training with cane providing auditory feedback  CG:  Gait training with cane without auditory feedback | Pressure sensor + auditory indicator  Walking cane; Sensor placed at base of cane  Auditory indicator around waist | Baseline  Immediately  after intervention | EG CG  Balance and Gait:  Single limb support  of the affected limb: ↑* -  Gait speed: ↑* -  Muscle activation:  Gluteus medius: ↑* -  Vastus medialis oblique: ↑* -  Weight-bearing:  Vertical peak force of the  cane (%Body weight): ↑* ↑ | Uneven demographics  No follow-up retention assessment  Inpatient therapy delivered alongside intervention |
| Nanhoe-Mahabier et al. 2012  (Holland) | To investigate  the effect of  vibrotactile biofeedback on trunk sway in PD. | n = 20  EG: n = 10  Mean age: 59.3±2 years  Male/Female:  8/2  CG: n = 10  Mean age: 58.6±2.5 years  Male/Female:  8/2 | 1 session of vibrotactile feedback  EG:  Balance and gait training with vibrotactile feedback  CG: Balance and gait training without vibrotactile feedback | 2 x IMUs + headband feedback stimulator  Belt device; Circumference of trunk level L1-L3  Headband; Circumference of forehead with vibrotactile sensors | Baseline  Immediately  after intervention | EG CG  Balance:  Centre of Mass (CoM)  sway angle (M/L): ↑* ↓  CoM sway angle (A/P): - -  Sway angular velocity (M/L): - -  Sway angular velocity (A/P): ↑* -  Gait:  Centre of Mass (CoM)  sway angle (M/L): ↑* -  CoM sway angle (A/P) - -  Sway angular velocity (M/L): - -  Sway angular velocity (A/P): - - | Very brief intervention  No follow-up retention assessment  Relatively high pre-intervention balance scores |
| Sungkarat et al. 2011  (Thailand) | To investigate whether auditory biofeedback to promote symmetrical weight distribution would improve balance and gait in people with stroke. | n = 35  EG: n = 17  52±7.17 years  Male/Female:  12/5  CG: n = 18  54±11.18 years  Male/Female:  12/6 | 3 weeks of auditory feedback  EG:  Balance and gait training with auditory feedback  CG:  Balance and gait training without auditory feedback | Pressure sensors + shoe wedge  Paretic shoe; Weight bearing sensor  Non-paretic shoe; footswitch (determines time in swing phase) and shoe wedge | Baseline  Immediately  after intervention | EG CG  Balance:  BBS: ↑ ↑  Single limb support  of the affected limb: ↑* ↑  Loading on paretic leg  during stance: ↑* ↑  Gait:  Gait speed: ↑* ↑  Step length: ↑ ↑  TUG: ↑ ↑ | No follow-up retention assessment  Lack of sub-group analysis for chronicity since onset of stroke |

↑ = Significant Improvement; ↓ = Regression; - = no significant changes; * = Significant difference between groups;
